# Individual Cow Recognition Based on Ultra-Wideband and Computer Vision

**DOI:** 10.3390/ani15030456

**Published:** 2025-02-06

**Authors:** Aruna Zhao, Huijuan Wu, Daoerji Fan, Kuo Li

**Affiliations:** 1 School of Electronic Information Engineering, Inner Mongolia University, Hohhot 010021, China; 32256022@mail.imu.edu.cn (A.Z.); fandaoerji@imu.edu.cn (D.F.); 32256003@mail.imu.edu.cn (K.L.); 2 Inner Mongolia Key Laboratory of Intelligent Communication and Sensing and Signal Processing, Inner Mongolia University, Hohhot 010021, China

**Keywords:** location, cow identification, precision farming

## Abstract

This study’s primary goal is to use computer vision and ultra-wideband (UWB) localisation techniques to automatically mark numerals in cow photos. In order to accomplish this, we created a UWB-based cow localisation system that involves installing tags on cow heads and placing several base stations throughout the farm. The system can determine the distance between each base station and the cow using wireless communication technology, which allows it to determine the cow’s current location coordinates. The study employed a neural network to train and optimise the ranging data gathered in the 1–20 m range in order to solve the issue of significant ranging errors in conventional UWB positioning systems. The experimental data indicates that the UWB positioning system’s unoptimized range error has an absolute mean of 0.18 m and a standard deviation of 0.047. However, when using a neural network-trained model, the ranging error is much decreased, with an absolute mean of 0.038 m and a standard deviation of 0.0079. The average root mean square error (RMSE) of the positioning coordinates is decreased to 0.043 m following the positioning computation utilising the optimised range data, greatly increasing the positioning accuracy. This study used the conventional camera shooting method for image acquisition. Following image acquisition, the system extracts the cow’s coordinate information from the image using a perspective transformation method. This allows for accurate cow identification and number labelling when compared to the location coordinates. According to the trial findings, this plan, which integrates computer vision and UWB positioning technologies, achieves high-precision cow labelling and placement in the optimised system and greatly raises the degree of automation and precise management in the farming process. This technology has many potential applications, particularly in the administration and surveillance of big dairy farms, and it offers a strong technical basis for precision farming.

## Simple Summary

In this paper, we propose a method for identifying individual cows based on computer vision and ultra-wideband localisation, since the majority of existing research can only identify the individuals in the current video or image and cannot link each individual in the video or image with the cow code used by the farm personnel. In this work, it is feasible to mark cow numbers in photos and track individual cows continuously by utilising ultra-wideband localisation technology in conjunction with the ability to distinguish individual cows in images. It provides a solid basis for later health monitoring, such as identifying cow behaviour and detecting cow heat, which supports farms’ intelligent management and accurate breeding.

## 1. Introduction

One of the main forces behind economic growth is a robust cattle sector. As herd sizes and farming practices change, it is particularly important to gather detailed information to increase the management effectiveness of dairy farms because animal behaviour can visually represent the physical state and health of the animals. Research has indicated a positive correlation between rumination time and milk production in early lactation cows and that rumination time monitoring is a reliable indicator of milk output [1]. Dairy cows are significantly impacted by heat stress, which can result in disorders such as lameness in addition to changing their eating and drinking habits, which lowers milk output and fertility [2]. Consequently, evaluating the impact of heat stress on a dairy herd requires paying close attention to the drinking and feeding habits of dairy cows [3,4]. Dairy cows’ respiration rates are also impacted by heat stress, and tracking these rates not only aids in evaluating the effects of heat stress but also efficiently diagnoses respiratory conditions and guarantees prompt treatment, both of which enhance dairy cow health and productivity [5]. Enhancing reproductive efficiency, maximising herd management, and boosting farm economic benefits are all significantly impacted by dairy cow oestrus detection. It is possible to guarantee that cows can conceive in an ideal reproductive state by precisely determining their oestrus period. This is important for the sustainability of dairy farming and the welfare of individual cows [6,7].

Precision dairy farming research, which is primarily based on two features of touch sensors and computer vision perception, has advanced significantly in recent years due to the rapid development of the Internet of Things (IoT) and artificial intelligence (AI) [8]. The activity level can be measured more precisely with touch sensor-based cow health monitoring technology [9].

In order to assist in the early prediction of diseases by gathering health-related data from cows, a real-time monitoring system was presented for cow rumination behaviour based on three-axis acceleration sensors and edge computing [10]. In order to improve the system’s performance, the ultra-wideband localisation technology is further integrated, which leads to a more precise and dependable identification of several behaviours, including eating, rumination, and resting [11]. A wearable cow nose ring was also created, which can record and classify cow behaviour data in real time, increasing the technology’s usefulness. These data serve as a foundation for examining the nutritional and ruminant habits of cows, offering a practical way to keep an eye on their health [12]. An individual cow feed intake measurement system was created by another researcher using gravity sensors and implemented on a commercial farm. The experimental results demonstrated that the system’s feed intake error was kept within 120 grammes, and the system’s performance in a commercial setting demonstrated its viability and efficacy in real-world applications [13].

Because computer vision technology is inexpensive and simple to use, it has emerged as a useful tool for tracking the wellbeing and health of dairy cows [14,15]. By using optical flow and inter-frame differencing techniques to follow the cows’ mouth region, one study was able to detect rumination behaviour with an accuracy of 89.12% [16]. By extracting gait and texture traits and fusing them with 3D processing techniques, another study was able to identify cows with an accuracy of 84.2% [17]. The outcomes of cow health monitoring are more accurate when machine learning techniques are used. With a proper recognition rate of 92.03%, a study using the Mean Shift algorithm successfully enhanced the monitoring accuracy of rumination behaviour by accurately tracking the mouths of cows [18]. Additionally, researchers developed a model with an accuracy rate of 82.37% for identifying oestrus in dairy cows using LOGISTIC regression and support vector machine (SVM) models [19]. Some experiments used convolutional neural networks with residual neural networks to identify individual cows with an accuracy of 94.53% [20]. Another study effectively raised the accuracy of individual cow recognition to 98.5% by cascading the DeepOtsu algorithm with the EfficientNet method [9].

We make sure that we can fully understand the health status of cows in real time and offer a strong guarantee for the effective management of farms and the health and wellbeing of cows in order to develop intelligent farms, realise refined farming, and commit to lowering labour costs while greatly enhancing the efficiency and accuracy of cow health monitoring. To transform farm management, we have investigated and evaluated individual cow identification technologies. Current visual target tracking methods depend on the system automatically assigning identification; however, this is incompatible with the farm management system’s requirement that ear tags serve as an individual’s unique identity, and the two are frequently at odds. We offer a novel solution to this problem by combining visual target detection and tracking methods with localization approaches to identify individual cows globally. By replacing the automatically generated IDs with the actual cow identification, this method can improve farm management’s accuracy and efficiency. We can optimise the management process of dairy farms and increase economic efficiency by integrating this cutting-edge technology, which guarantees that every cow is accurately paired with its own unique identification.

## 2. Materials and Methods

**Figure 1:**
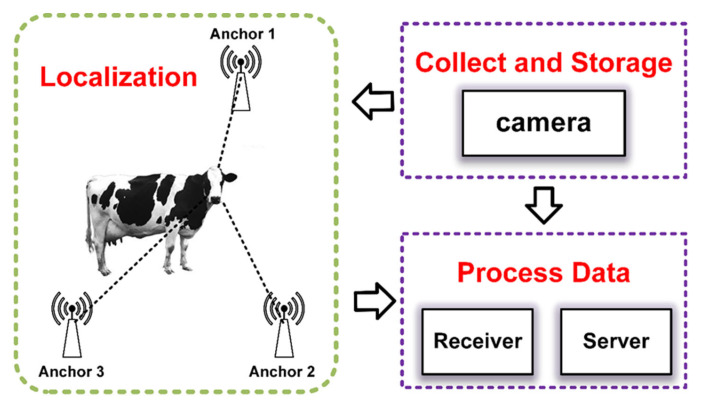
Deployment of experiments.

A tag, three base station nodes, a receiver, and a server make up the entire data collecting system, which also serves as a full localisation data processing system. A 5 V battery powers the tag, which is worn on the cow’s head. A camera (Huawei Ltd., Shanghai, China; the shooting parameters are 60 frames per second and 1080p) is also required for this experiment in order to take real-time pictures of the cows and transmit the information to the server side for image processing. Launched on 20 August 2024, the experiment lasted for two hours. We carried out image acquisition once every minute during this period, keeping the localization system operational the entire time. In Tongliao City, Inner Mongolia, we used one cow from a particular villager for data acquisition. The base station was hung from posts surrounding the cow pasture, the camera was kept off the edge of the cow pasture, and the tags and batteries were tied together in a plastic bag on the cow’s head. Figure 1 depicts the experiment’s precise deployment.

### 2.1. Hardware Designs

The STM32L051 (STMicroelectronics Ltd., Geneva, Switzerland) microcontroller serves as the positioning system’s central processing unit and is in charge of data processing, interruption management, and system timing control. The STM32L051 chip is especially well suited for systems that need to run continuously for extended periods of time, like sensor networks and Internet of Things devices, because of its exceptional low-power characteristics and support for multiple power saving modes. In addition to extending battery life, the low-power feature guarantees system reliability and continuity over extended use. It has a 32 MHz ARM Cortex-M0+ CPU, 64 KB of flash memory, 8 KB of SRAM, and a typical maximum operating current of 1–5 mA. It runs between 1.8 and 3.6 V.

The system uses the DW1000 (Decawave Ltd., Dublin, Ireland) chip, which is specifically made for UWB positioning, to achieve high-accuracy range measuring. In an outdoor setting, the system’s effective range in this trial was 30 m, with a ranging accuracy of 0.18 m. Furthermore, the system’s outstanding multi-tag processing capabilities have been demonstrated by its ability to support up to six tags simultaneously while in operation. By regularly sending and receiving UWB signals between the two DW1000 chips, the range measurement may be accomplished because the DW1000 chip not only supports data transmission but also has the capability to receive. The technology achieves high-precision (accuracy of about 10 cm) localisation by precisely determining the distance between the two chips by logging the time stamps of the transmitted and received signals and using particular range algorithms. The DW1000 chip is the ideal option for short-range positioning applications due to its exceptionally low power consumption and potent anti-jamming capabilities. It has a 290 m range and can achieve precise placement. The chip guarantees steady functioning in spite of multipath effects and signal interference, making it especially appropriate for usage in complex environments like factories, warehouses, and other locations with high reflections and sources of interference.

**Figure 2:**
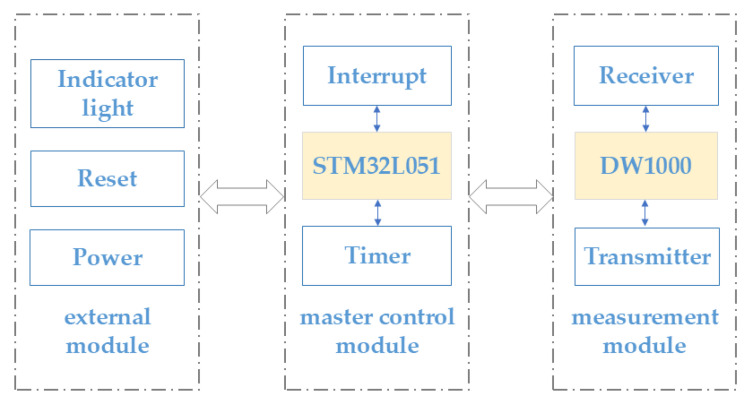
Block diagram of UWB positioning system hardware.

The system’s pushbuttons and indicators enable the user to view the system’s operational status in real time in addition to its primary locating function. The overall block diagram is displayed in Figure 2. The buttons enable users to conveniently start, stop, or reset the system, and the indicator lights give feedback on the current operating status or error messages through various colours or flashing modes, allowing users to intuitively understand whether the system is operating normally and to make adjustments or maintenance in time. In order to guarantee the system’s dependability and effectiveness in a range of application scenarios, its design fully accounts for high-precision positioning, low power consumption, anti-jamming capabilities, and user interaction.

### 2.2. Localization Algorithm

**Figure 3:**
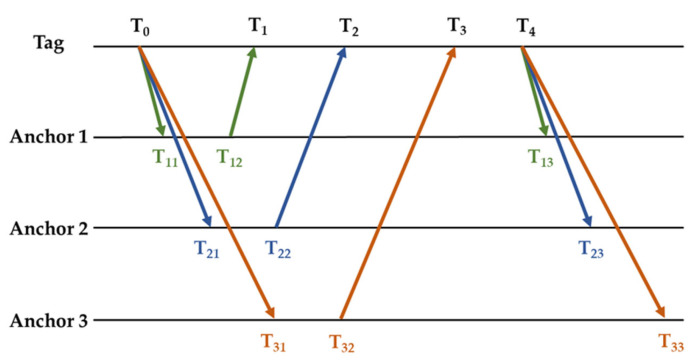
Positioning algorithms.

In order to minimise the tag’s power consumption and extend its lifespan, this study uses an optimised positioning technique. Figure 3 illustrates the precise flow of this algorithm. By successfully lowering the amount of tag messages, the algorithm’s main goal is to lower power consumption and boost system efficiency. In Figure 3, *T*_0_ and *T*_4_ denote the tag’s signal sending time, and *Ti* (*i* = 1, 2, 3) denotes the tag’s reception time of each base station’s signal. The base stations’ time stamps are also captured during the particular range procedure.

In this case, *T_i_*_1_ and *T_i_*_3_ indicate the times at which anchor 1 receives the signal and broadcasts it, respectively. Each base station in our system will wait a predetermined amount of time after receiving a signal before responding, and a whole ranging process takes roughly 100 ms. Since a high ranging frequency is not necessary for this experiment, we intentionally set up a 5 s pause between each ranging operation before starting the subsequent ranging task. The precise distance between each base station and the tag can be calculated from these timestamps using the bilateral bidirectional ranging (DS-TWR) technique [21]. In particular, the following equation determines the distance d_1_ between the tag and base station 1:

(1) $$d_{1}=\frac{\left(T_{1}-T_{0}\right)*\left(T_{13}-T_{12}\right)-\left(T_{12}-T_{11}\right)*\left(T_{4}-T_{1}\right)}{\left(T_{1}-T_{0}\right)+\left(T_{13}-T_{12}\right)+\left(T_{12}-T_{11}\right)+\left(T_{4}-T_{1}\right)}*c$$

where c is the light speed. Similarly, the following equation is used to determine the distances *d_2_* and *d_3_* between the tag and base stations 2 and 3, respectively:

(2) $$d_{2}=\frac{\left(T_{2}-T_{0}\right)*\left(T_{23}-T_{22}\right)-\left(T_{22}-T_{21}\right)*\left(T_{4}-T_{2}\right)}{\left(T_{2}-T_{0}\right)+\left(T_{23}-T_{22}\right)+\left(T_{22}-T_{21}\right)+\left(T_{4}-T_{2}\right)}*c$$

(3) $$d_{3}=\frac{\left(T_{3}-T_{0}\right)*\left(T_{33}-T_{32}\right)-\left(T_{32}-T_{31}\right)*(T_{4}-T_{3})}{\left(T_{3}-T_{0}\right)+\left(T_{33}-T_{32}\right)+\left(T_{32}-T_{31}\right)+(T_{4}-T_{3})}*c$$

The trilateral localisation method can then be used to further calculate the tag’s precise coordinates after the distances from the three base stations have been determined. Equation (4) can be established assuming that anchor 1’s coordinates are (*x_i_*, *y_i_*) and the tag’s coordinates are (*x*, *y*):

(4) $$\left\{\begin{array}{c} d_{3}^{2}={(y_{3}-y)}^{2}+{(x_{3}-x)}^{2}\\ d_{2}^{2}={(y_{2}-y)}^{2}+{(x_{2}-x)}^{2}\\ d_{1}^{2}={(y_{1}-y)}^{2}+{(x_{1}-x)}^{2} \end{array}\right.$$

The label’s location can be ascertained by solving this equation, enabling high-precision real-time positioning.

This optimised positioning algorithm is ideal for application scenarios that demand high-precision positioning and long-term operation because it not only increases positioning accuracy but also efficiently decreases the number of communications between the tag and the base station, significantly lowers power consumption, and extends the tag’s service life. 

### 2.3. Neural Network Filtering Model

Even though UWB devices typically use high-precision crystal oscillators, or crystals, to generate clock signals, manufacturing flaws in crystals are inevitable. These flaws, along with the effects of temperature changes, supply voltage fluctuations, and other factors, cause tiny variations in the crystal’s frequency, which cause the clock to drift. With continued usage of the apparatus, this clock’s internal component instability will progressively build up and eventually result in clock offset inaccuracies. We must adjust for the mistake in the initial measurement data in order to acquire more accurate localisation findings, which are essential for ensuring the accuracy of individual identification.

There are several mistakes in the localisation findings and the image coordinate determination during the cow recognition step. We made the decision to modify the localisation error in order to guarantee the precision of individual cow identification and avoid the interference of intricate error components. This study suggests a backpropagation (BP) neural network-based error compensation technique as a result. We gathered static range data from 1 to 20 m (10 different data distributions per metre) in advance to use as input data for the BP neural network. This approach guarantees the high accuracy and dependability of cow identification by providing optimal ranging results.

**Figure 4:**
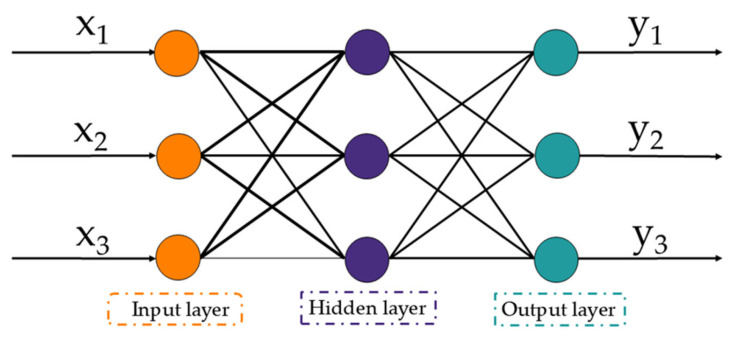
BP neural network structure: x is input and y is output.

Figure 4 illustrates the BP neural network, a traditional multilayer perceptron structure with input, hidden, and output layers. Both forward and backward propagation of information and errors are part of the network’s training process. As the neural network’s initial layer, the input layer is in charge of taking in external inputs and relaying them to the hidden layer, which is the second layer, during the forward propagation process. After receiving data from the input layer and processing it nonlinearly using an activation function, the neurones in the hidden layer send the processed data to the output layer, which is the third layer. The final prediction is produced by the output layer.

The backpropagation algorithm determines the error and modifies the connection weights of each layer in the neural network using the gradient descent approach when there is a difference between the intended and actual output. Through the output layer, the hidden layer, and finally the input layer, the error is backpropagated. Until the error is brought down to a predetermined tolerance level or a predetermined number of trainings and time is reached, this forward propagation and backpropagation procedure will keep going.

This procedure enables the BP neural network to obtain highly precise localisation by gradually optimising the distance measurements and removing the impacts of clock drift and other defects.

### 2.4. Transmission Transformation and YOLO Detection

Yolo detection of cows in the image is the first step in the experiment [22]. The Yolo v5s model was employed in this work and has a mean average precision of 92.6%, after which the perspective transform is used to achieve the unification of the image’s coordinates with the actual coordinates. One essential tool in computer vision is the perspective transform, which alters an image’s point of view to mimic a shift in the observer’s position. A 3 × 3 transformation matrix that transfers points from the original image plane to the new plane is used to achieve this. If there is a transmission transformation *f* from space P to space Q, then Equation (5) determines that for each vector a*(x,y,z)* in P to the image f(a) = b*(u,v,w)* in Q, where Equation (6) is the transmission matrix for the transmission transformation f. In actuality, the transmission transform of a space can be realised by automatically computing this matrix by entering the four points (although these four points in the image were selected at random, they cannot be on the same straight line; instead, they need to be picked based on the actual needs) in the original image and their locations in the corresponding four points in the transformed image.

(5) $$\left\{\begin{array}{c} u=c_{11}x+c_{12}y+c_{13}z\\ v=c_{21}x+c_{22}y+c_{23}z\\ w=c_{31}x+c_{32}y+c_{33}z \end{array}\right.$$

(6) $$C=\left[\begin{array}{ccc} c_{11} & c_{12} & c_{13}\\ c_{21} & c_{22} & c_{23}\\ c_{31} & c_{32} & c_{33} \end{array}\right]$$

**Figure 5:**
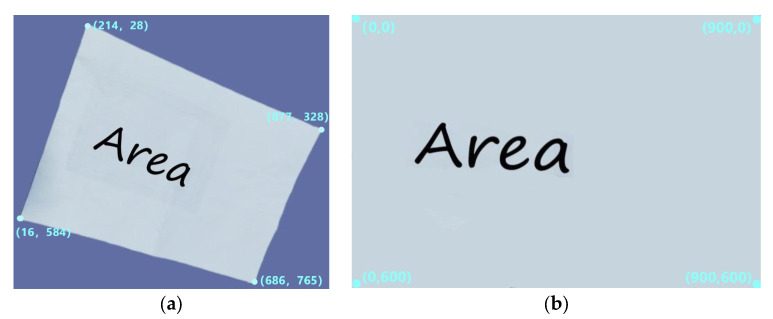
Examples of transmission transformations: (**a**) original image; (**b**) transformed image.

Figure 5a displays the original image, which has 900 × 780 pixels. The target area of interest, a rectangle that was initially 30 cm by 20 cm in size, is the white region in the centre of the picture. The target area seems distorted in the picture as a result of the dismissing angle. We must first determine the coordinates of the target region’s four corners in the original image, as indicated in Figure 5a, which are (214, 28), (877, 328), (16, 584), and (686, 765), in order to recreate the target region’s original mould and extract it into a new image. 

The coordinates of the target region’s four corners are then defined as follows in the new image: (0, 0), (900, 0), (0, 600), and (900, 600). This indicates that the new image created by the target region that was extracted has a dimension of 900 × 600 pixels. Using the perspective transformation function, we can finish transforming the entire image using the four points that are known to correspond to before and after the transformation. In Figure 5b, the altered image is displayed. Furthermore, the new image’s pixel size is 900 × 600, matching the target region’s actual size of 30 × 20 cm. We set the scale factor to 30 in order to achieve the correlation between the image’s size and the actual coordinates. By performing this process, we are able to accurately match the image’s size to its actual size while also restoring the target region’s original appearance.

### 2.5. Evaluation Indicators

We selected the RMSE as the primary evaluation metric in order to thoroughly evaluate the localisation system’s accuracy and dependability. A quantitative indicator of the positioning findings’ correctness, this metric is calculated by taking the square root of the average of the squares of the differences between the expected and actual values. The positioning error is reduced and the positioning system performs better when the RMSE value is decreased. Equation (7) is used for calculation:

(7) $$\mathrm{RMSE}=\sqrt{\frac{1}{n}\sum_{i=1}^{n}{{\left(x_{i}-\hat{x_{i}}\right)}^{2}+(y_{i}-\hat{y_{i}})}^{2}}$$

where n is the number of samples, ($\hat{x_{i}},\;\hat{y_{i}}$) is the ith sample’s actual value, and (*x_i_, y_i_*) is the ith sample’s measured value.

## 3. Results

### 3.1. Error Compensation Model Effect

Using the previously gathered range data, we initially trained the BP neural network to increase the localisation system’s accuracy. We separated the 200 data points in the dataset into 70% training data and 30% testing data. Because the experiment’s dataset was so small, we deliberately selected fewer hidden layer nodes to prevent overfitting brought on by an overly complex model. Furthermore, in order to exclude the training model with the best performance, we systematically compared the number of hidden layer nodes with various configurations. Considering fewer datasets, we can guarantee the model’s optimal generalisation performance.

**Figure 6:**
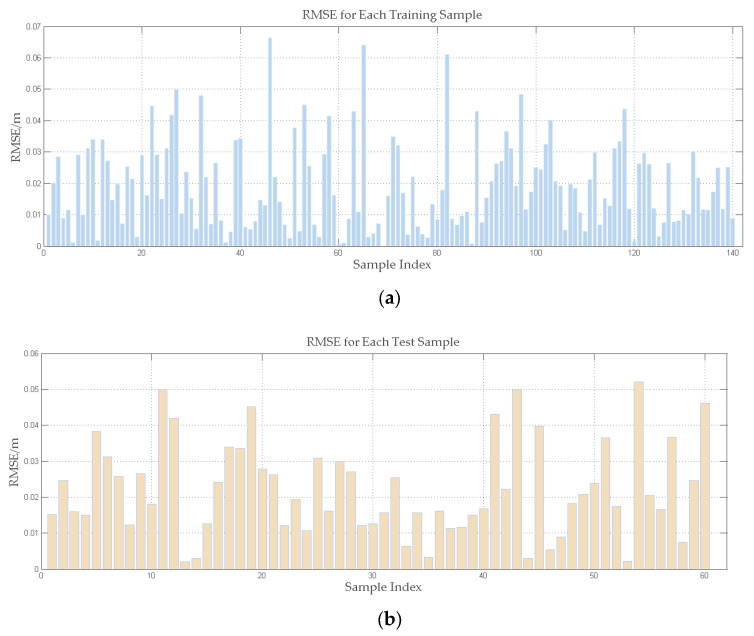
Results for the training and test sets: (**a**) the RMSE of the training set; (**b**) the RMSE of the test set.

The comparison analysis’s findings are detailed in Table 1, which demonstrates that setting the number of hidden layer nodes to six optimises the model’s performance. In particular, the RMSE for the training set is currently 0.0238 and the RMSE for the test set is 0.0256. Figure 6 illustrates this outcome. The model’s excellent potential for error compensation is demonstrated by its high prediction accuracy on both the training and test sets. We have developed an efficient error compensation model as a result of this procedure.

### 3.2. Static Positioning Test

We performed a static localisation test in a cowshed to assess the model’s localisation accuracy in authentic settings. Three base stations were positioned uniformly throughout the test site, which was initially chosen inside the cowshed, and their coordinates were noted. The distance between each tag and the three base stations was then measured after the tags were positioned at three randomly chosen spots in the centre of the test site.

We next computed the localisation coordinates using the processed data after filtering the obtained distance data using an error compensation model based on BP neural networks. The average RMSE of the three test points following filtering is 0.043 m, according to Table 2, which compares the localisation results before and after filtering. According to the results of Table 2, the localisation accuracy has greatly increased following processing by the error compensation model, and it can meet the actual use requirements in cow farms.

### 3.3. Individual Identification Results

**Figure 7:**
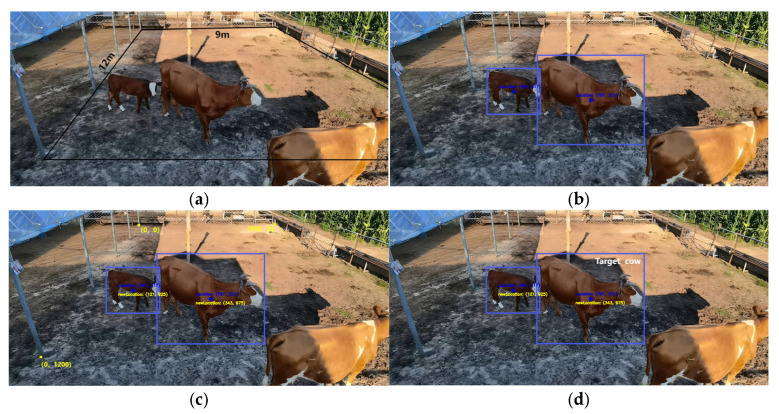
Process of individual identification: (**a**) schematic of selected areas; (**b**) results of yolo testing; (**c**) coordinate conversion results; (**d**) target identification results.

As seen in Figure 7a, a camera was employed to capture the pertinent image at the edge of the cowshed in a 12 m × 9 m experimental area. Initially, we labelled every cow in the experimental region, performed YOLO detection on this image, and noted the cows’ coordinates. The detection results are displayed in Figure 7b. To precisely locate the cows, we set the centre point of the detected bounding box as the image’s cow coordinates. Two cows are identified in all, one of which is a target cow and the other is not.

We then performed a transmission transformation using the corresponding positions of the four points that were transformed (the transformed coordinates matched the real coordinates in centimetres) after selecting four points at random from the image (in this paper, we selected four random points on the border of the selected area, and the coordinates of these points are known). Figure 7c illustrates how we used the transmission transformation to unify the picture coordinates with the real coordinates and convert the image’s cows’ coordinates to the real coordinates.

Lastly, we compare the coordinates from the tag with the coordinates in the picture to find the target cow. It should be mentioned that the image indicates the cow’s body’s centre position, whilst the tag determines the cow’s head position. Therefore, in order to perform an exact comparison, a margin of error is set. Adult dairy cows served as the study’s subjects. Since calves and adult cows are typically kept apart on large farms, the situation of calves was not discussed in this study; instead, it concentrated solely on adult cows. Adult cows typically measure 2.5 m in length, with 1.2 m separating the head from the middle of the body. To guarantee inclusivity and measurement accuracy, we set the margin of error at 1.4 m for actual observations, including for the potential impact of natural postures like cows’ lowered heads. Additionally, the corrected range must not be greater than the detected bounding box. It can be confirmed that the cow is the one listed by the matching tag if the positioning result falls within the image’s cow’s coordinates. The experiment’s findings are displayed in Figure 7d, and the target cow was located.

In conclusion, this experiment confirms the viability and efficacy of the chosen algorithm while correctly identifying the target cows.

## 4. Discussion

### 4.1. Performance of Individual Recognition Technology

To increase animal welfare and breeding efficiency, dairy cows’ physiological and behavioural characteristics must be accurately monitored [23]. In order to study typical cow activities, cows have been tracked using a UWB positioning system, in which the cow wears a positioning device to identify the region of the barn in which it is located [24]. Nevertheless, the health tracking of a particular cow and animal identification are not possible with the dairy cow monitoring equipment currently in use. It is vital to physically search the barn for a specific cow when a health problem is found in that cow. The cost of labour is higher on larger farms. In certain situations, cow cover may make it impossible to identify the cows, even if they are equipped with ear tags to help with rapid identification. The cow can be quickly positioned in the cow yard, labour expenses can be decreased, and the annoyance of an ear tag being obstructed can be avoided by wearing a positioning device and integrating it with computer vision technology.

The cow code in the video at the start of the target tracking is currently unable to automatically recognise and associate each individual in the video with the cow code used by the ranch personnel [25]. This is because the current machine vision-based technology primarily uses the target recognition method first to find the individual and continuous tracking. In reality, it is necessary to track each cow separately.

Each cow in the pasture can have its precise coordinates obtained by the UWB localisation system in this study. Following the translation of plane coordinates to image coordinates, each cow’s code can be marked in the image. This resolves the issue of integrating cow coding with the image-based individual cow identification approach. Precision farming is made possible by this method, which also greatly increases farm managers’ productivity [26,27]. Accurate cow tracking was achieved in the trials presented in this study by successfully integrating computer vision and UWB localisation technology to tackle the problem of recognising cow code in photographs. The system can effectively correlate to each cow’s unique coding in complicated image contexts thanks to the precise spatial localisation support offered by UWB localisation technology and sophisticated computer vision algorithms, guaranteeing real-time cow tracking and localisation. This method can continuously function in dynamic surroundings in addition to effectively identifying cow codes in static photos, which provides a strong basis for real-time recognition in video streams. More significantly, this technique offers a technical guarantee for precision farming, which can capture and analyse each cow’s behavioural data in real time, including activities, feeding, resting, and many other aspects. The data collected over time can also support farming management with in-depth behavioural analysis. Furthermore, the use of this technology can support the development of the farming sector in the direction of intelligence and personalisation, which is crucial to improving the sustainability and productivity of animal husbandry [28], as well as the health management and production efficiency of dairy cows [29,30].

### 4.2. Further Perspectives on Individual Recognition Technology

The realisation of precise and intelligent dairy cow husbandry still faces numerous obstacles. Even if the research findings presented in this work have effectively completed the planned experimental tasks and met the initial goals, there are still a lot of pressing issues that need to be resolved in order to move on. Additionally, this study has many shortcomings that merit more thorough investigation and examination.

(1) The chosen region is tiny since the camera range for this experiment must cover the entire chosen area; therefore, a camera with a longer focal length will be required as the experimental apparatus in the future.
(2) The experimental object that was chosen is small: only one cow was chosen for this experiment; in the real-world scenario, if there were more cows on the cattle farm, the measurement error would be higher. Therefore, in order to improve the experiment’s practicality, the experimental object should be increased.
(3) The cows in this experiment were positioned in two dimensions without taking Z-axis coordinates (height direction) into account. Stated otherwise, the experiment was evaluated only on the basis of planar position, disregarding the cows’ height. The precision of the experimental results, however, can be impacted by the height disparities between the cows when there are several of them.
(4) There are drawbacks to the power supply design. The label’s power supply battery in this experiment is a 5V lithium battery, which requires replacement every few days. The power supply’s design should be further enhanced for better use in real-world situations.

## 5. Conclusions

In this study, we successfully implemented a cow localisation system at a cow farm using a design based on the DW1000 chip. At first, the system’s range inaccuracy was 18 cm. To increase localization accuracy, we optimised the BP neural network to lower the range error to 3.8 cm, and the localization result’s root mean square error was only 4.3 cm. We use transmission transformation to unify the picture coordinates with the actual coordinates, using the centre point of the YOLO detection frame in the image as the cow’s coordinates. Individual cows were then identified by comparing the localization findings with the actual coordinates. The algorithm’s ability to correctly recognise the cow number in the picture is demonstrated by the experimental findings, which strongly support the behavioural monitoring and precise management that follow.

Relying only on manual monitoring of each cow’s health state on large farms not only uses a lot of human resources but also runs the danger of errors and omissions in observations. Accurately recognising cow numbers from photos is a technological advancement that completes computer vision-based cow identity validation and establishes a strong basis for future cow tracking and health monitoring. The precision and efficiency of monitoring will be significantly increased if this technology is combined with sophisticated tools like heat detection and cow behaviour identification, resulting in genuinely intelligent and accurate farming.

## Figures and Tables

**Table 1:** RMSE corresponding to different hidden layers.

Number of Hidden Layers	Training Set RMSE/m	Test Set RMSE/m
4	0.027	0.031
5	0.024	0.029
6	0.024	0.026
7	0.025	0.027
8	0.025	0.029
9	0.024	0.028

**Table 2:** Test point localization results.

Test Points	Real Coordinates/m	Before Filtering Coordinates/m	After Filtering Coordinates/m
1	(1.6, 3.6)	(1.655, 3.673)	(1.626, 3.628)
2	(3, 5)	(3.052, 5.08)	(3.032, 5.034)
3	(4, 6.5)	(4.065, 6.605)	(4.0281, 6.533)

## Data Availability

Data are contained within the article.
